# Methods and design for the ADAPT study: Application of integrateD Approaches to understanding Physical activity during the Transition to emerging adulthood

**DOI:** 10.1186/s12889-020-08484-0

**Published:** 2020-03-31

**Authors:** Matthew Y. W. Kwan, Pallavi Dutta, Steven R. Bray, Denver M. Y. Brown, John Cairney, Genevieve F. Dunton, Jeffrey D. Graham, Amanda L. Rebar, Ryan E. Rhodes

**Affiliations:** Department of Family Medicine, McMaster University, 100 Main Street, DBHSC 5th Floor, Hamilton, Ontario L8P 1H6 Canada

**Keywords:** Adolescence, Emerging adulthood, Transition, Physical activity, M-PAC, Ecological momentary assessment

## Abstract

**Background:**

The overarching objective of the study is to obtain a comprehensive understanding of the salient factors predicting changes in physical activity (PA) during adolescents’ transition into emerging adulthood. Using the Multi-Process Action Control model as our guiding framework, we will examine how implicit and explicit psychological processes along with regulatory practices impact PA change during this major life transition. Additionally, we will use a real-time data capture method called Ecological Momentary Assessment to further investigate how environmental and contextual factors, and momentary psychosocial influences effect PA patterns across this dynamic life stage.

**Methods:**

The ADAPT study is a 4-year project comprised of two interrelated studies. Study I is a large prospective cohort study that will invite all grade 11 students across one large school board (a total of seven secondary schools) to participate by completing an online questionnaire. Using a cluster randomization approach, a subset of students from each school will be invited to participate in Study II, whereby participants will wear an accelerometer and complete Ecological Momentary Assessments 5 times a day over a 7-day study period. For both studies, following baseline assessments, there will be three annual follow-up assessments approximately 12 months apart.

**Discussion:**

The current study represents one of the largest longitudinal cohort studies examining PA and its determinants and associated consequences among adolescents transitioning out of high school into emerging adulthood. Findings from this study will provide a much more in-depth understanding of how and why changes in PA behaviour occur across this first major life transition.

## Background

Physical inactivity has become a major public health concern [1–3]. Indeed it is well established that the lack of physical activity (PA) engagement is associated with increased risk of several chronic diseases, directly contributing to high health care costs [4–6]. However, it also carries significant indirect and social costs such as time off work, lost productively, and psychosocial problems (e.g., low self-esteem, low sense of competence, delinquency) [7–10]. In terms of total estimated energy expenditure, trends suggest that PA peaks during the adolescent period, with significant declines in participation thereafter [10, 11]. In Particular, the transition from adolescence into emerging adulthood has been identified as a specific point for which steep declines in PA occur [12–20].

The transition into emerging adulthood is often considered the first major life transition [21–24], representing a period for which individuals acquire greater autonomy and some characteristics of adulthood without fully reaching the milestones that socially define contemporary adulthood [24]. The notable life event within this transition period is high school graduation. The specific transition out of high school is found to be associated with major changes in lifestyle behaviours, including PA [12]. Specifically, the literature has been very consistent in showing that PA behaviours are higher when individuals are in high school compared to the year following high school graduation [12–19]. There are a number of notable limitations that exist within the aforementioned body of evidence and require further investigation. First, few studies have followed participants from high school, instead relying on participants to retrospectively report PA during their high school years despite having already graduated. Second, few studies have used objective physical activity measures such as accelerometers to follow individuals over time. Third, in comparison to behavioural studies examining PA declines, few studies have investigated how changes in social cognitive factors relate to the changes in PA behaviours. Finally, little is known about how changes in social, environmental, and contextual factors may be influencing behaviour change during this transition.

In order to inform and develop effective long-term population-level interventions to target PA decline during the transition from adolescence to emerging adulthood, a better understanding of the underlying socio-environmental and psychological processes related to changes in PA behaviour is essential. Existing research that has investigated how psychosocial factors relate to PA behaviours around this life stage has been primarily based on social cognitive theories such as Theory of Planned Behaviour [25] and Social Cognitive Theory [26]. These theories suggest that behaviour is largely a function of an evaluation of expected behavioural outcomes and the confidence to perform it, manifesting through the conscious formation of an intention to enact the behavior [25–29]. Accordingly, while primacy has been given to these antecedent factors, few studies have focused on the non-conscious, social, and environmental influences. Moreover, little consideration has been given to within-subject changes (i.e., variability) and the influences of momentary (day-to-day, hour-to-hour) fluctuations in PA-related cognitions. As a result, despite the transition into emerging adulthood being highly dynamic, we have little knowledge about how changing contextual environments, variable affective or motivational states, and shifting opportunities may be influencing changes in PA during this life stage.

Action process-based models are becoming increasingly popular, particularly the application of integrative approaches to include post-intentional psychological constructs to bridge the intention-behaviour gap [30–34]. The Multi-Process Action Control (M-PAC) framework [35] represents one recent example. Described as a harmonized model based on well-established antecedents of PA behaviour and the integration of regulatory constructs to better explain PA behaviours. What makes M-PAC particularly unique is that it also allows for less conscious influences such as habit and identity impacting PA behaviours at the maintenance phase. M-PAC positions PA to be processes-based with PA being influenced by three central constructs: reflective processes to denote the consciously deliberated expected consequences of performing PA and the act of behavioral performance (i.e., intention formation); regulatory processes that include behaviours or cognitions that people enact to translate their intentions into physical activity behavior (i.e., self-regulated behavioural action); and reflexive processes that represent impulsive or less reasoned constructs that influence action control most often through learned associations and are triggered through particular circumstances and stimuli (i.e., habits and identity). To date, however, few studies have attempted to examine the M-PAC model in its totality.

It is also important to acknowledge that associations between psychosocial factors and PA have generally been examined at the between-person level only. That is, most studies have assessed an aggregated form of motivation and behaviour (i.e., a general rating of what you think about how active you would like to be over a specified period of time, and how much PA you engaged in over that period) [36] . The primary limitation of this approach is that an aggregated or gestalt perception about a behaviour assumes and considers motivation or perceptions to be stable across time. Emerging evidence based on several diary-based studies suggest that young people’s cognitions, and subsequent behaviours tend to vary over the course of a day [36–39]. Understanding the impact of within-person variations as it relates to how an individual may be thinking and its influence of how they behave is important, particularly if we can understand momentary cognitions using an existing theoretical framework such as the M-PAC model. The current protocol paper will describe the proposed procedures and methodology for a study aimed at addressing some of these aforementioned limitations – the ADAPT study: Application of integrateD Approaches to understanding Physical activity during the Transition to emerging adulthood.

## Methods

### Study design

The ADAPT study is comprised of two interrelated studies. Study I begins as a school-based study, using a prospective cohort design to follow a group of individuals during the broader transition from adolescence to emerging adulthood. Specifically, it is questionnaire-based, aiming to recruit a robust sample of as many of the grade 11 students across one large school board (a total of seven secondary schools), serving a socially and economically diverse metropolitan centre located in Southern Ontario. A subset of Study I participants will be invited to participated in Study II. Using a cluster randomization approach, two or three classes from each of the seven schools will be selected and subsequently invited to participate in Study II**.** This related study will involve more intensive social cognitive and PA assessments using ecological momentary assessments or EMA. For both studies, following baseline assessments, there will be three annual follow-up assessments approximately 12 months apart – the idea being to follow participants as they complete their final years at high school and first 2 years following graduation.

### Recruitment Strategy & Study Protocol

#### STUDY I

Baseline assessments will take place in classes, and all grade 11 students at each of the participating schools (ages 14–17) will be asked to voluntarily participate in this study. On average, the school board estimates that there are 300 grade 11 students in each school, and we expect that 80% will agree to complete the baseline questionnaire assessment. Having established a strong partnership with the school board, each of the seven secondary schools have agreed to participate in the larger study, and each with a nuanced and individualized recruitment strategy. Across each school, letters of information and consent forms will be sent out to students and parents approximately 1 week in advance of the school data collection date. The data collection date will fall within a five-week data collection period, mutually agreed upon between the school and study team. Data collection will occur during the first period of classes at each school, whereby all students will be invited to use the first 20 min of class to complete the online questionnaire. Each teacher will be informed in advance by school administrators and will provide students with the link to the questionnaire. Research assistants will be on site for all data collection dates, attending each classroom to provide further background to the study, answer any student questions, and/or to troubleshoot any potential problems with the online questionnaire. Participation is voluntary for students, but each student will be provided a $10 gift card if they choose to participate.

The vast majority of students will still be in high school during the initial follow-up (approximately 12-months later), thus the data collection period will again take place at the secondary schools. However, given that the final two data collection periods will take place following high school graduation, attempts will be made to re-contact participants via e-mail or other contact information provided to the study team. During each follow-up period, participants will be required to complete an online questionnaire, receiving a $10 gift card for each time they participate.

#### STUDY II

A subset of Study I participants will be asked to participate in this related study with more rigorous and intensive assessments. Students attending two or three selected classrooms within each school will be invited to participate in Study II. To be eligible, students must be (a) enrolled into Study I, and (b) own a smartphone. In addition to completing the baseline questionnaire, participants in Study II are being asked to wear a wrist-based accelerometer and to complete EMA prompts via their smartphone five times a day over a seven-day study period. In an attempt to capture the most representative sample at baseline, two to three English classes will be randomly selected from each school (all students are required to take English in their grade 11 year). Specifically, one university, college, and vocational stream class will be selected at each school in an attempt to capture different post-high school trajectories (i.e., entering postsecondary education, joining the workforce, etc.).

A research assistant will attend these select classes, providing all students with the purpose and study requirements of Study II. Pre-populated study packages including an accelerometer and a personalized code for an EMA app will be distributed to students interested in participating. Participants will be then be asked to download an EMA app developed for this study called Lumedi (designed and developed by Webility Solutions Inc.), and to enter the unique code provided. Immediately following this, they will be instructed to put the accelerometer on their non-dominant hand and to wear it for as much as they can over the next week (with the exception of showering and participation in water-based activities), and to answer as many of the five prompts on each day. Given the high participant burden, participants will be provided with $100 gift card for participation at baseline and each subsequent follow-up period.

### Measurements

#### Socio-demographic factors

Participants will be asked to respond to demographic questions that include items to self-report age, grade, gender, ethnicity, parental education, and residential postal code (used as a proxy measure of household income).

#### Self-reported physical activity

The International Physical Activity Questionnaire – Short Form (IPAQ-SF) will be used to measure PA [40, 41]. Specifically, participants will be asked to respond to 3 items and indicate, during the last 7 days, on how many days they did 1) vigorous physical activities, 2) moderate physical activities, and 3) walked for at least 10 min at a time. If they indicate that they participated in an activity for one or more days, then they will be asked to indicate how much time (hours and/or minutes) they usually spent on one of those days participating in that activity.

#### Physical activity cognitions

The physical activity cognitions measures included are largely based on the M-PAC model [35]. Below are the specific measures included:

*Affective and Instrumental Attitudes* will be measured using 6 items and presented on a 7-point bipolar scale [42]. Each item will be prefaced with the stem “*Over the next 4 weeks, being physically active, for me, will be*”. The affective component will be captured by three items: unpleasant-pleasant, boring-fun and unenjoyable-enjoyable. The instrumental component will be captured by the three items: harmful-beneficial, bad-good and useless-useful.

*Behavioural Intention* for engaging in PA will be measured using a single item measure commonly used in the PA domain [43]. Participants will be asked to rate the extent to which the following statement is likely: “*I intend to do at least 150 minutes per week of moderate-to-vigorous physical activity*”. Response options will range from 1 (*extremely unlikely) to* 7 (*extremely likely)*.

*Task and Barrier self-efficacy* will be measured by adhering to recommendations from Bandura (2006) for assessing self-efficacy [44]. That is, each item will be prefaced with the stem “*How confident are you in your ability to* …” with responses ranging from 0 (*Not Confident*) to 10 (*Completely Confident*). For task self-efficacy, participants will be asked to rate their confidence to perform 7 items relating to physical activity behaviour such as *“to engage in PA*”, “*to engage in sports*”, “*to jump, hop, and skip*” and “*to balance*”. For barrier self-efficacy, participants will be asked to rate their confidence in being physically active if “*the weather was very bad*”, their “*schedule conflicted with [their] activity session*” and “*even if [they] felt overwhelmed with things to do*”.

*Perceived Opportunity* and *Perceived Capability* for engaging in physical activity will be measured by asking participants to indicate their agreement with 6 items pertaining to their perceived opportunity and perceived capability (i.e., confidence and/or control) for engaging in regular physical activity [45]. Each item will be rated on a 7-point *adjectival* scale ranging from 1 (*Strongly Disagree*) and 7 (*Strongly Agree*). Perceived opportunity items include “*If I really wanted to do regular physical activity over the next 4 weeks, I would have a chance to do so”*, *“I lack the opportunity to do regular physical activity over the next 4 weeks, even if I were really motivated to do so”*, and *“There are places where I can do physical activity around me if I had to*”. Perceived capability items include *“I possess the skills to do regular physical activity over the next 4 weeks if I wanted to”*, “*I have the physical ability to do regular physical activity over the next 4 weeks if I wanted to*”, and “*I am confident that I am capable of engaging in regular physical activity if I had to*”.

*Action Planning will be measured using the Behavioral Regulation for Physical Activity scale* [46] *which consists of 6 items that are rated on a 5-point Likert scale ranging from 1 (Strongly Disagree) to 5 (Strongly Agree). Example items include “I kept track of my physical activity over the last month”, “I set short-term (daily or weekly) goals for leisure-time physical activity last month” and “I made regular plans concerning ‘when’, ‘where’, ‘how’, and ‘what’ kind of physical activity I did last month”.*

*Social Support:* A modified version of the Multidimensional Scale of Perceived Social Support (MSPSS) will be used to assess social support [47]. Specifically, the 12-item measure includes three subscales, addressing a different source of support by either (a) family, (b) friends, and (c) significant others. Participants will be asked to what extent they agree with statements such as “*I get the emotional help and support I need from my family*”, “*My friends really try and help me*”, and “*There is a special person in my life whom I can share my happiness and sadness*”. Responses will be measured on a 7-point *adjectival* scale ranging from 1 (*Strongly Disagree*) to 7 (*Strongly Agree*). Previous research has demonstrated that the MSPSS has good internal and test-retest reliability as well as good construct validity [48].

*PA Identity* will be measured using the Exercise Identity scale [49], which asks participants how exercise is related to their concept of self. The Exercise Identity scale contains 5 items that are rated on a 7-point *adjectival* scale ranging from 1 (Strongly Disagree) to 7 (Strongly Agree). Participants will rate their agreement with statements such as “I consider myself as someone who is physically active” and “I would feel at a real loss if I were forced to give up physical activity”.

Self-Reported Habit for engaging in PA will be measured by the Self-Report Habit Index [50], asking participants about the extent to which engaging in physical activity is a habit for them. The Self-Report Habit Index contains 4 items that are rated on a 7-point Likert scale ranging from 1 (Strongly Disagree) to 7 (Strongly Agree). Each item will be prefaced with the stem “Regular physical activity is something …” . Example items include “… I do automatically” and “… I start doing before I realize I’m doing it”.

#### Psychosocial measures

*Trait Self-Control* will be measured using 5 items from the brief version of the Trait Self-Control Scale [51]. Each item will be rated on a 5-point *adjectival* scale ranging from 1 (*Not at all like me*) to 5 (*Very much like me*). The specific items include “*I have a hard time breaking bad habits*”, “*I am lazy*”, “*Fun sometimes keeps me from getting work done*”, “*I am able to work effectively toward long-term goals*”, *“I often act without thinking through all the alternatives*”, and “*I am good at resisting temptation*”.

*Fatigue* will be measured using the Chalder Fatigue Scale [52] which consists of 11 items that are rated on a 4-point *adjectival* scale ranging from 1 (*Less than usual*) to 4 (*Much more than usual*). Example items include “*Do you have problems with tiredness?*” and “*Do you have difficulties concentrating?*”

*Flourishing* will be measured using the Flourishing Scale [53] consisting of 8 items that are rated on a 7-point Likert scale ranging from 1 (*Strongly Disagree*) to 7 (*Strongly Agree*). Example items include “*I lead a purposeful and meaningful life*”, “*My social relationships are supportive and rewarding*”, and “*I am optimistic about my future*”.

*Self-Esteem* will be measured using a modified version of the Rosenberg Self-Esteem Scale [54] which measures global self-worth through statements relating to both positive and negative feelings relating to self. Included will be the 5 items that are associated with positive feelings towards self, “*On the whole, I am satisfied with myself*”, “*I feel that I have a number of good qualities*”, “*I am able to do things as well as most other people*”, “*I feel that I’m a person of worth, at least on an equal plane with others*”, and “*I take a positive attitude towards myself*”. Reponses will be rated on a 4-point *adjectival* scale ranging from 1 (*Strongly Disagree*) to 4 (*Strongly Agree*).

*Coping Resiliency* will be measured using 2-items that are rated on a 5-point scale ranging from 1 (*Poor*) to 5 (*Excellent*). Each item will be prefaced with the stem “*In general, how would you rate*”, and specific items include “*Your ability to handle unexpected and difficult problems (a family or performance crisis)*” and “*Your ability to handle day-to-day demands in your life (work, family responsibilities*)”. These items are consistent with those used in the Canadian Campus Wellbeing Survey [55].

*Sense of Belonging* will be measured using 3 items from the Sense of Belonging scale [56] rated on a 4-point *adjectival* scale ranging from 1 (*Strongly* Disagree) to 4 (*Strongly* Agree). The original scale is a 10-item measure that contains items relating to the domains of commitment, engagement and connectedness. The questions adapted for this study includes the three items from the engagement domain. Each item will be prefaced with the stem “*Thinking about your school, how much would you agree or disagree with the statements below*”, and the specific items include “*I feel comfortable at my school*”, “*I feel like I am part of my school*”, and “*I am supported at my school*”.

#### Ecological momentary assessment measures

Participants in Study II will be required to complete a series of 5 EMA prompts each day over the 7-day study period. Given the intensive sampling strategy, very brief questionnaires were developed to reduce participant burden (i.e., surveys designed to take one-to-two minutes to complete). The EMA questions will assess self-reported contextual information on current activity, current affective and feeling states, and measures of acute psychological processes consistent with M-PAC.

*Context-Specific Measures:* Each prompt sent to participants will include questions to obtain contextual information on three domains: (a) what participants are currently doing, (b) where participants currently are, and (c) with whom they are currently accompanied by. These items have been used in previous research, and have been found to be valid measures [57, 58].

*Affective and Feeling State* will be measured using the Hardy and Rejeski Feeling Scale [59]. Assessed on an 11-point scale, the 2 items ask “How do you feel right now” assessing acute arousal (− 5 Tired to + 5 Energetic) and valence (− 5 Very Bad to + 5 Very Good).

Reflective Process will be assessed using 2 items. First a single item of task motivation asking “How motivated are you to do physical activity right now?”. Responses will range from 0 (*Not motivated at all*) to 10 (*Extremely motivated*). Second, we will include a question of whether or not participants intend to do 10+ minutes of PA sometime within the next few hours (*yes/no*).

*Regulatory Process* will be assessed using 3 items*.* Two action control based questions will ask: “Could you do 10+ minutes of PA in the next hour even if you get busy?” and “Could you do 10+ minutes of PA sometime within the next few hours even if you get tired?”. Participants will respond on a 5-point scale from 1 (*I know I cannot*) to 5(*I know I can*). An additional item of self-control will also be included, asking: “If I were tempted by something right now, it would be very difficult to resist”. This item will be measured on 7-point scale ranging from 1 (*Not true)* to 7 *(Very true).*

*Reflexive Process* will be assed using a single item to capture habit-like behaviours. The question asks “Currently, I am doing something I would normally be doing on a typical day at this time”, with responses on a 5-point Likert scale from 1 (*Strongly disagree*) to 5 (*Strongly agree*).

#### Accelerometer-based physical activity

Participants in Study II will be asked to wear an ActiGraph GT9X Link on their non-dominant wrist for 7 complete days. This device will be set to collect data in 3 axes at a sampling rate of 30 Hz. Participants will be instructed to wear the device as much as possible while awake and sleeping, removing it only for prolonged water exposure. Wear time will be determined using the Troiano (2007) criteria, with any period ≥60 min of consecutive 0 counts flagged as non-wear [60]. Days that the accelerometer was dropped off and picked up will be excluded from analyses. PA data will only be analyzed for participants who meet the minimum wear time criteria (≥10 h on ≥3 days). Data will be analyzed using ActiGraph’s CentrePoint for daily total activity counts, average activity counts per minute, and daily steps.

### Data analysis

For Study I, Structural equation modeling (SEM) and mixed effects modeling (MEM) will be used to address the primary research objectives of the current study. SEM will be used to test the M-PAC framework, modeling and identifying key influences related to PA pre-transition, and to examine the changes in latent (i.e. unobserved constructs) and observed variables through the transition into emerging adulthood. MEM or growth curve modeling will be used to examine the influences of individual factors on PA behaviours accounting for individual students nested within their classrooms and nested within schools. Our models will include indicators of SES and social cognitive factors (i.e., mediators), being entered as time-varying independent variables, while other socio-demographic factors (moderators) will be entered in our models as time-invariant independent variables.

For Study II we will use *time-varying effects modeling* (TVEM) to investigate the associations between momentary cognitions and subsequent PA [61]. TVEM models examine changes in the associations between intensively sampled environmental-contextual or momentary social cognitive factors as it relates to PA behaviours over time. This method can accommodate the unequal spacing of observations and unequal assessments across participants, typical of studies using EMA. Modeling time effects with intensive longitudinal data captures complex patterns of variation, and generally produces confidence regions rather than the *p*-values associated with hypothesis testing.

## Discussion

The current study represents one of the largest longitudinal cohort studies examining PA and its determinants and associated consequences among adolescents transitioning out of high school into emerging adulthood. Findings from this study will provide a much more in-depth understanding of how and why changes in PA behaviour occur across this first major life transition. The overall study will also advance theory by testing a relatively new model inclusive of stable and dynamic environmental and psychosocial factors in the prediction of behavioural patterns, and how variations in these factors within the day impact daily PA. An important feature of this study is its established partnership and sampling from across an entire school board in Southern Ontario, enabling us to investigate how behavoural patterns, and its determinants and consequences may differ across socioeconomic groups and different trajectories following high school graduation. As a school-based study, we do anticipate a number of challenges. For example, despite the strong ongoing support by the school board, there are always challenges with school engagement. Our strategy is to work with each individual school as a partner in the research process, inviting them to contribute ideas for data collection. Another and potentially greater challenge will be to maintain participant engagement across the 4-year study duration. Attrition has at times been challenging with this population, [16, 17] given that many of these individuals have not been exposed nor have had the opportunity to fully appreciate the research process. To mitigate attrition, we will ensure that both the survey tool and EMA prompts are developed not to overburden participants. In addition to the initial follow-up taking place at schools as the first part of a class, we are also offering a generous $100 compensation for Study II participants. Overall, the results from the current study will help to inform the basis for future intervention work during the transition from adolescence through emerging adulthood; identifying how interventions can be designed to effectively target the right outcomes, for the right group, and at the right time.

## Data Availability

Not applicable.

## Abbreviations

PA: physical activity
MVPA: moderate-to-vigorous physical activity
EMA: ecological momentary assessment
HWCDSB: Hamilton-Wentworth Catholic District School Board
MSPSS: Multidimensional Scale of Perceived Social Support
